# Experimental and theoretical studies on antituberculosis activity of different benzimidazole derivatives

**DOI:** 10.1016/j.heliyon.2025.e42674

**Published:** 2025-02-12

**Authors:** Suna Kızılyıldırım, Berfin Sucu, Muhammed Tilahun Muhammed, Senem Akkoç, Tuba Esatbeyoglu, Fatih Ozogul

**Affiliations:** a Cukurova University, Faculty of Pharmacy, Department of Pharmaceutical Microbiology, Adana, Türkiye; b Cukurova University, Institute of Science and Technology, Department of Biotechnology, Adana, Türkiye; c Süleyman Demirel University, Faculty of Pharmacy, Department of Pharmaceutical Chemistry, 32260, Isparta, Türkiye; d Süleyman Demirel University, Faculty of Pharmacy, Department of Basic Pharmaceutical Sciences, 32260, Isparta, Türkiye; e Bahçeşehir University, Faculty of Engineering and Natural Sciences, Istanbul, 34353, Türkiye; f Gottfried Wilhelm Leibniz University Hannover, Institute of Food and One Health, Department of Molecular Food Chemistry and Food Development, Am Kleinen Felde 30, 30167, Hannover, Germany; g Cukurova University, Faculty of Fisheries, Department of Seafood Processing Technology, Adana, Türkiye; h Cukurova University, Biotechnology Research and Application Center, Adana, Türkiye

**Keywords:** Tuberculosis, Benzimidazole, Antituberculosis, Molecular docking, Molecular dynamics simulation

## Abstract

Tuberculosis (TB) continues to be one of the deadliest infectious diseases with a rapid increase in multidrug-resistant cases. The discovery of new agents against tuberculosis is urgently needed. Thus, the research article focuses on the antituberculosis activity of a series of benzimidazolium compounds. The antituberculosis activities of compounds including benzimidazole core (**7a-h**) against *Mycobacterium tuberculosis* H37Rv strain were tested *in vitro* using the BACTEC MGIT 960 system. The concentrations of benzimidazole compounds were adjusted to range from 0.25 to 4 μg/ml. The antituberculosis interactions of the compounds were investigated by molecular docking and molecular dynamics simulation. The results revealed that only benzimidazolium salt **7h** showed antituberculosis activity at MIC value of 2 μg/ml although the other compounds showed no antituberculosis activity. The docking data revealed that **7h** could bind to InhA thus indicating its inhibition potential on the enzyme. Molecular dynamics simulation exhibited that **7h** formed a stable complex with the enzyme and was able to remain inside the binding region of the enzyme. Besides, the pharmacokinetic and drug-likeness properties of the compounds were assessed through computational approaches. The compounds exhibited drug-like properties. Consequently, **7h** could be a good candidate for the development of new TB drugs.

## Highlights

• Antituberculosis activity of benzimidazolium compounds against *M. tuberculosis* H37Rv strain was tested.
• The compound **7h** showed antituberculosis activity.
• The binding potential of active compound (**7h**) to InhA was examined using molecular docking and MD simulation.
• The docking study showed that **7h** had the potential to bind to the enzyme.
• The compound **7h** could be a good candidate for the treatment of tuberculosis.

## Graphical abstract

**Figure:**
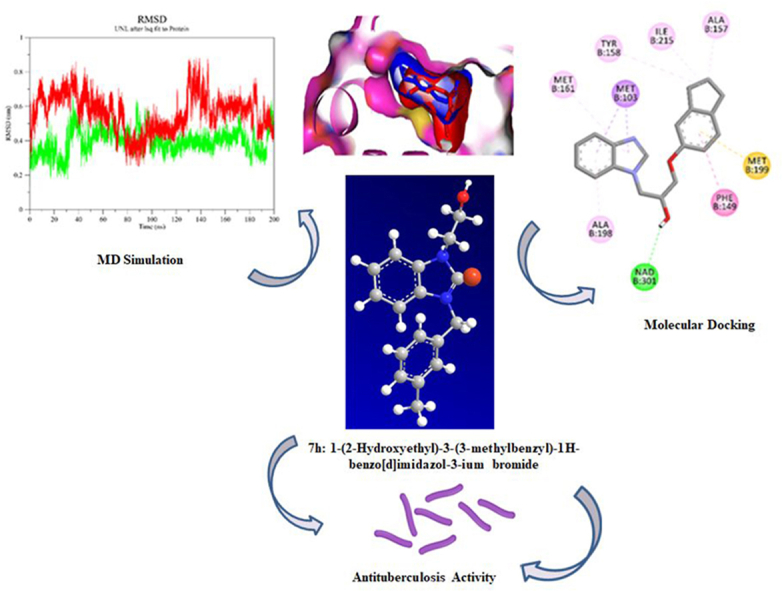

## 1 Introduction

Tuberculosis (TB) is a serious infection caused by *Mycobacterium tuberculosis* and one of the top 10 causes of death worldwide [[1], [2], [3]]. The disease affects about 10 million individuals globally, and 1.5 million of those people pass away from it each year [4,5]. The report of WHO 2022 states that over 1.4 million persons with TB-related deaths and over 0.18 million deaths from HIV-TB coinfection occurred [6].

The lungs are the initial location of the infection, and it spreads from there through the circulatory and lymphatic systems to secondary sites including the bones, joints, liver, and spleen [7]. The treatment of TB is a demanding, intricate, time-consuming, and incredibly difficult task [8,9]. Besides, TB is difficult to treat because it entails the administration of isoniazid (INH), rifampicin (RIF), pyrazinamide (PZA), and ethambutol (EMB) in combination for the first two months, followed by the continuation of INH and RIF for an additional 4–7 months [10]. Long-term multidrug TB treatment has been demonstrated to overburden patients with a heavy dosage of pills and adverse effects, which significantly lowers inpatient compliance and adherence to the medicine. This increases the potential risk for the development of drug-resistant TB strains [11].

Drug-resistant tuberculosis exists in almost all countries of the world, making global tuberculosis treatment even more difficult [12]. Drug-resistant tuberculosis requires more than 20 months of therapy, and its cure rate is lower [13]. Although drugs such as bedaquiline, delamanid and pretomanid are used in the clinic for the treatment of drug-resistant TB, *M. tuberculosis* strains have also developed resistance to these drugs [14]. This situation has led researchers to work on developing new antituberculosis agents. Effective treatment and early, accurate diagnosis of tuberculosis are essential to tuberculosis control [15]. In the treatment of TB, there is an urgent need for the trial and development of new, simplified treatment, safe, long-acting antituberculosis drugs with a low probability of resistance development.

INH is one of the original drugs used to treat tuberculosis [16] and it inhibits cell wall synthesis by inhibiting enzymes involved in cell wall synthesis. Many enzymes discovered in these layers, including InhA, are prospective targets for the design and development of novel anti-TB medicines [17].

InhA is a member of the NADH-based enoyl-ACP reductase enzyme family found in *M. tuberculosis*. This enzyme catalyzes the NADH-specific reduction of 2-*trans*-enoyl-ACP in the elongation cycle of mycolic acid, the major component of the mycobacterial cell wall in the fatty acid synthase type II (FAS II) pathway [18,19]. Therefore, InhA has long been used as a target for the development of antitubercular drugs. InhA does not have an ortholog in humans. It has a conserved active site which makes it unique from the other targets in *M. tuberculosis*. Its deep binding pockets in the active site give the opportunity of the design and development of various small molecule inhibitors. Hence, InhA remains as an attractive target for the design and development of antitubercular agents [20,21].

Benzimidazole is a heterocyclic compound that is extensively utilized as an organic synthesis building block [22]. The compounds having benzimidazole nucleus have a crucial function in medicinal chemistry research, and many compounds containing benzimidazole have beneficial biological features like antiviral, anti-inflammatory, and anti-HIV effects [7]. Benzimidazole nucleus is important as an antibacterial agent due to its heteroatom and electron-rich aromatic ring structure [23]. The antituberculosis activity of compounds having benzimidazole core is available in many literatures [24,25].

Different hypotheses have been put forward regarding the antituberculosis mechanism of action of benzimidazole-derived compounds. Benzimidazoles are structural isosteres of purines and thus can inhibit the biosynthesis of nucleic acids and proteins by competing with heterocycles, leading to the death of bacterial cells. They can effectively inhibit bacterial topoisomerases [26]. Benzimidazolium compounds can also prevent the GTPase activity of *M. tuberculosis* filamentous temperature-sensitive Z and septum formation [27,28]. Therefore, this study aims to examine the antituberculosis activity of some benzimidazolium derivatives and to investigate their mechanisms of action to identify a new antituberculosis candidate agent.

## 2 Materials and methods

### 2.1 Synthesis of benzimidazolium salts

**Fig. 1:**
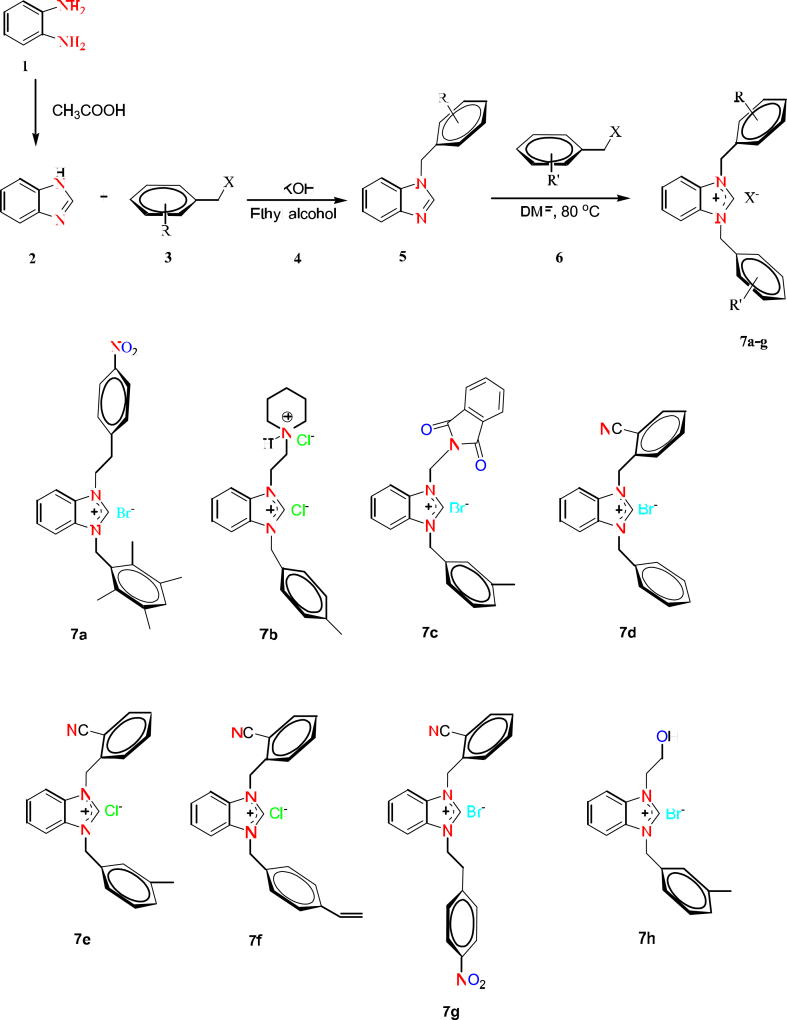
Chemical structures of synthesized compounds.

Benzimidazole was synthesized from orthophenylenediamine. Then, potassium hydroxide (KOH) was added to the solution of benzimidazole in ethyl alcohol. Following this, aryl/alkyl halide (1 mM) was added to over this reaction mixture. Benzimidazolium salts were synthesized from N-alkylbenzimidazole (1 mM) and aryl halides in DMF. Following compounds, 1-[2-(4-nitrophenyl)ethyl]-3-(2,3,5,6-tetramethylbenzyl)-1H-benzo[d]imidazole-3-ium bromide (7a) [29], 1-(4-methylbenzyl)-3-(2-(piperidinium-1-yl)ethyl)-1H-benzo[d]imidazole-3-ium dichloride (7b) [30], 1-(*N*-phthalimidomethyl)-3-(3-methylbenzyl)benzimidazolium bromide (7c) [31], 1-(2-methylbenzonitrile)-3-benzylbenzimidazolium bromide (7d) [32]; [33]; [34], 1-(2-methylbenzonitrile)-3-(3-methylbenzyl)benzimidazolium chloride (7e) [35], 1-(2-cyanobenzyl)-3-(4-vinylbenzyl)-1H-benzo[d]imidazole-3-ium chloride (7f) [29], [36], 1-(2-cyanobenzyl)-3-[2-(4-nitrophenyl)ethyl]-1H-benzo[d]imidazole-3-ium bromide (7g) [29]; [37] were synthesized according to mentioned above procedure. 1-(2-Hydroxyethyl)-3-(3-methylbenzyl)-1H-benzo[d]imidazole-3-ium bromide (7h) [38] was synthesized and fully characterised by ^1^H NMR, ^13^C NMR, IR, and HRMS. The open structures of compounds are given in Fig. 1.

### 2.2 Antituberculosis activity against *M. tuberculosis* H37RV strains

The study was carried out at the Tropical Diseases Research and Application Center, Cukurova University (Adana Regional Tuberculosis Laboratory). The *in vitro* tuberculostatic activity of the synthesized benzimidazolium salts (**7a-h**) against the *M. tuberculosis* H37Rv strain was investigated. The antituberculosis activity was performed according to the protocol used by Gallo et al. [39]. The antituberculosis activities of the benzimidazolium salts (**7a-h**) were tested *in vitro* using the BACTEC MGIT 960 system. The final drug concentrations for the BACTEC MGIT 960 system were 2.0 μg/ml for STR (streptomycin), 0.1 μg/ml for INH, 2.0 μg/ml for RMP (rifampin), and 2.5 μg/ml for EMB. The concentrations of benzimidazolium salts were adjusted to 0.25–4 μg/ml and tested in the semi-automated BACTEC MGIT960 device integrated with the EpiCenter System. MIC values were analysed every 18–24 h using the BD EpiCenter database compared to growth control tubes without benzimidazolium salts. The growth control and drug-containing inoculation tubes were put in a five-position drug susceptibility testing (DST) set carrier and inserted into the device as “unknown drugs.” The device marked the test set as complete when the growth control achieved a growth unit of 400. The results were considered as resistant if the growth unit of the drug-containing tubes exceeded 100 while the growth unit of the growth control was 400. The drug-containing tubes with growth unit values ≤ 100 were considered susceptible.

### 2.3 Molecular docking

The crystal 3D (dimensional) structure of InhA, which is a crucial target in antituberculosis drug design and discovery, was retrieved from the protein data bank (PDB). The protein structure utilized has a co-crystallized active ligand (1-(1H-benzo[d]imidazole-1-yl)-3-((2,3-dihydro-1H-inden-5-yl)oxy)propan-2-olinside) with a common benzimidazole ring to the synthesized compounds (PDB code: 6R9W) [40]. Molecular docking was performed with AutoDock Vina [41]. The molecular docking was performed as reported studies [42]. Thereafter, docking results were visualized and analysed with Biovia Discovery Studio [43].

### 2.4 Molecular dynamics (MD) simulation

MD simulation was performed with the GROMACS package to measure the stability of enzyme-compound complexes obtained from the docking [44]. The automated CHARMM general force field (CGenFF) was utilized to parametrize the compounds [45]. The enzyme and compound topologies were prepared as reported in previous studies. After all, the necessary processing was done, MD simulation was run for 200 ns [46]. Finally, RMSD (root mean square deviation), RMSF (root mean square fluctuation), Rg (radius of gyration), and ligand hydrogen bond plots were drawn from the MD simulation trajectories through qtgrace and analysed [47].

### 2.5 MMPBSA computation

Binding energy and contributing energies of InhA-compound complexes were computed through the MMPBSA (molecular mechanics Poisson-Boltzmann surface area) method. MMPBSA computations were performed by using g_mmpbsa [48]. The binding and contributing energies were computed through this method. The figures obtained were used to compare the binding affinities of the compounds and the role of the contributing energies in achieving the binding. Moreover, the energy obtained was broken down into per-residue energies and interpreted.

### 2.6 In silico ADMET

Computational pharmacokinetics properties of the synthesized compounds were predicted with Discovery Studio 3.5 [49]. In addition, the drug-likeness of the compounds was estimated with a SwissADME server [50]. PSA-2D (polar surface area-2 dimensional), AlogP98 (atomic logarithmic partition coefficient), BBB (blood-brain barrier) permeability, PPB (plasma protein binding), and Ames toxicity were predicted through Discovery Studio [51]. The drug-likeness of the compounds was also assessed by testing their compliance with Lipinski's rule of 5 (RO5) through the SwissADME server.

## 3 Results and discussion

### 3.1 Antituberculosis activity

*M. tuberculosis* is an important threat to human health. It is very difficult to treat due to the development of resistance to existing antibiotics. Therefore, it is necessary to develop new antituberculosis agents for the eradication of TB.

Benzimidazoles have been reported to have low toxicity and are highly effective against many pathogenic strains. The heterocyclic ring system in benzimidazoles has been found in both naturally occurring and synthesized medicinal compounds; and has been a fundamental building block in the development of many marketed drugs [22].

In order to discover new agents effective against *M. tuberculosis*, eight different benzimidazolium salts (**7a-h**) were investigated against *M. tuberculosis* H37Rv reference strain using the MGIT960 system. The **7a-g** derivatives of the benzimidazole compounds showed no antituberculosis activity. **7h** Benzimidazole compound (1-(2-hydroxyethyl)-3-(3-methylbenzyl)benzimidazolium bromide) showed antituberculosis activity against *M. tuberculosis* with MIC value of 2 μg/ml**.** The lack of antituberculosis activity of other compounds may be attributed to structural differences. Fig. 1 shows that both of the substituents attached to the nitrogen atoms of the benzimidazole nucleus of compounds **7a-g** consist of cyclic structures. The compound showing the highest effect in this study (7h) contains a cyclic structure at one position and a 2-hydroxyethyl group at the other position. This shows that the 2-hydroxyethyl as substituent plays an important role on *M. tuberculosis* rather than the benzyl, 2-cyanobenzyl, 3-methylbenzyl, 4-methylbenzyl, 4-vinylbenzyl, 2-(4-nitrophenyl)ethyl, 2,3,5,6-tetramethylbenzyl, N-phthalimidomethyl, and 2-(piperidinium-1-yl)ethyl substituents. The 3-methylbenzyl substituent in the structure of compound **7h** is also found in compounds **7c** and **7e**. The fact that these two compounds are inactive like other compounds (**7a**, **7b**, **7d**, **7f**, and **7g**) supports the idea that the 2-hydroxyethyl group is effective in increasing the activity on *M. tuberculosis*.

In our previous study, it was determined that benzimidazolium salts prepared by binding different substituents at one and three positions of the benzimidazole ring did not show antituberculosis activity. This study shows that the positions to which substituents are attached play an important role on activity [52]. In another own study with benzimidazoles revealed that compound S2 (1-(N-methylftalimide)-3-(4-methylbenzyl) benzimidazolium bromide) showed antituberculosis activity, S2 binds with InhA and can exert its antituberculosis effect by inhibiting it [53]. In addition, it was reported in many investigations that different benzimidazole compounds had antituberculosis activity against both reference strains and clinical *M. tuberculosis* strains. According to reports, 1H-benzo[d]imidazole derivatives exhibited antitubercular activity *in vitro* at a nanomolar range of concentrations [54]. In a different study, it was revealed that 2,5,6-trisubstituted benzimidazoles had strong anti-TB activity [55].

It was found that compound **7b**, which had a high cytotoxic effect on different cancerous cell types, had a toxic effect on the healthy human embryonic kidney cell line (HEK-293T) and human epithelial normal lung cell line (Beas-2B) with IC_50_ values of 25.89 and 18.10 μM, respectively [30]. Compounds **7d** and **7e** were tested against the HEK-293T cell line in our earlier studies and its IC_50_ values were determined as 64.06 ± 5.13 and 53.25 ± 3.70 μM, respectively [32,35]. The inhibition effects of compound **7d** on biofilm formation, pyocyanin, elastase, and *Pseudomonas aeruginosa* PA01 were investigated. Compound **7d** appeared to reduce swarming motility and pyocyanin production in *P. aeruginosa* PA01 at the dose tested [33]. The toxic effect of compounds 7f and 7g on healthy embryonic kidney cell line was tested *in vitro* in our previous study [29]. Compounds **7f** and **7g** were found to have low toxic effects against the HEK-293T cell line with IC50 values of 92.71 ± 3.59 μM, and >100 μM, respectively.

Studies demonstrated that many benzimidazole compounds had antituberculosis activity, which is extremely promising for the treatment of TB [8,24,25,56,57]. In the near future, some benzimidazole compounds may become new candidates for the treatment of tuberculosis with clinical trials.

### 3.2 Molecular docking

**Fig. 2:**
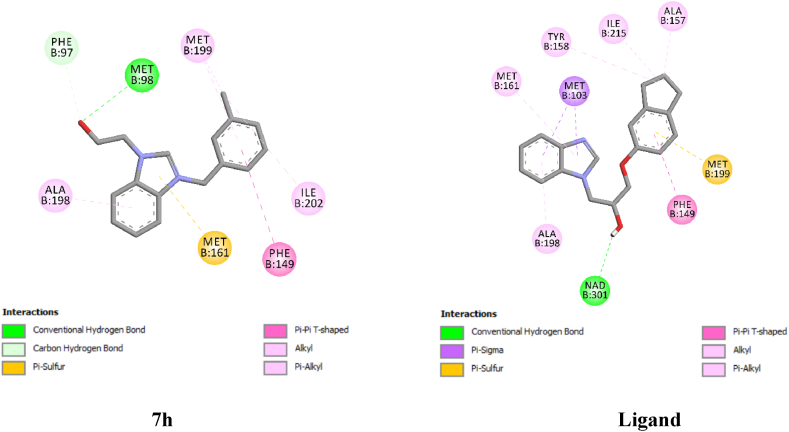
The two-dimensional binding profile of **7h** and the bound ligand with InhA (6R9W).

The molecular docking results demonstrated that the relatively active compound, **7h**, could bind to InhA. Compound **7h** bound to InhA with one conventional hydrogen bond (Met98), one carbon-hydrogen bond (Phe97), one pi-pi (Phe149), and four pi/alkyl-alkyl interactions (Ala198, Met199(2), Ile202). Similarly, the bound ligand showed good binding towards InhA with one conventional hydrogen bond (NAD301), two pi-sigma (Met103), one pi-sulphur (Met199), one pi-pi (Phe149), and five alkyl/pi-alkyl interactions (Ala157, Tyr158, Met161, Ala198, Ile215). The docking analysis implied that the bound ligand might bind slightly better than **7h** (Fig. 2).

The docking process was validated by re-docking the bound ligand onto InhA. Together with its good interactions, the binding was compatible with the previous experimental studies. The docking results revealed that the bound ligand interacted with InhA with NAD301, Met103, Phe149, Ala157, Tyr158, Met161, Ala198, Met199, and Ile215 residues (Fig. 2). A previous crystallographic analysis of InhA with the ligand inside it revealed interactions with Met103, Phe149, Ala157, Tyr158, Ala198, Met199, and Ile215 residues [40]. In another X-ray crystallographic study, a compound interacted with InhA at most of the binding residues detected in this computational study including NAD301 and Met161 [58].

There was an agreement with the study of Sullivan et al. [59] and this work in terms of some common binding points including a hydrogen bond with NAD [59]. Similarly, in an experimental study that investigated the binding of triclosan with InhA, most of the interactions in this study and a hydrogen bond with NAD were observed [60]. Moreover, other previous experimental studies gave interaction patterns that are like this computational study [61,62]. In short, the docking results were found to be compatible with previous experimental studies. After the docking process was validated in this way, a docking study of **7h** was pursued.

**Fig. 3:**
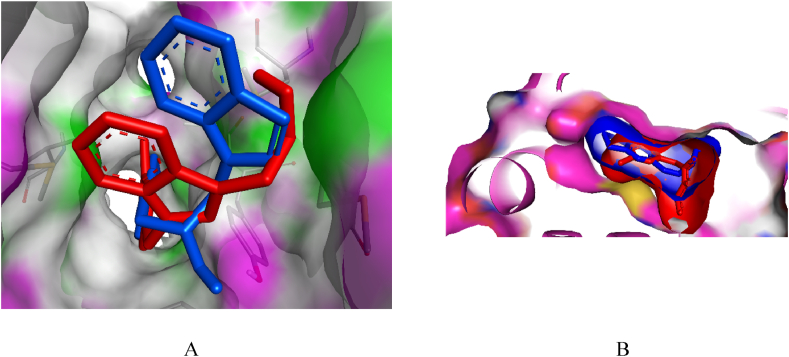
The relative orientations of compound 7h (in red) and the co-crystallized inhibitor (in blue) in the binding site of InhA. The protein coloration for A is as follows: Fluorescent pink-hydrogen bond donors (HBDs) and green-hydrogen bond acceptors (HBAs).

According to the molecular docking results, **7h** had interactions with InhA at Phe97, Met98, Phe149, Ala198, Met199, and Ile202 residues (Fig. 2). In the previous X-ray crystallographic structure determination, various ligands interacted with InhA at Phe149, Ala198, Met199, Ala201, and Ile202 residues [63]. In another experimental study, an inhibitor interacted with InhA at Phe97 [62]. Therefore, all the interactions detected in this study except the one at Met98 were observed in previous wet-lab studies. The molecular docking results were found to be compatible with the findings in the literature available. In addition to this, the binding pattern of **7h** has some level of similarity to the bound ligand. In this regard, the interactions at Phe149, Ala198, and Met199 residues are common to both compounds (Fig. 2). Along with these, the interaction of **7h** is slightly weaker than the bound ligand. The settling of compound **7h** was also like the settling of the co-crystallized inhibitor in the binding site of the enzyme (Fig. 3). To sum up, the molecular docking study revealed that **7h** has the potential to bind to InhA. Hence, the antituberculosis effect in the *in vitro* study might result from its potential to bind and thus inhibit the InhA enzyme.

### 3.3 MD simulation

**Fig. 4:**
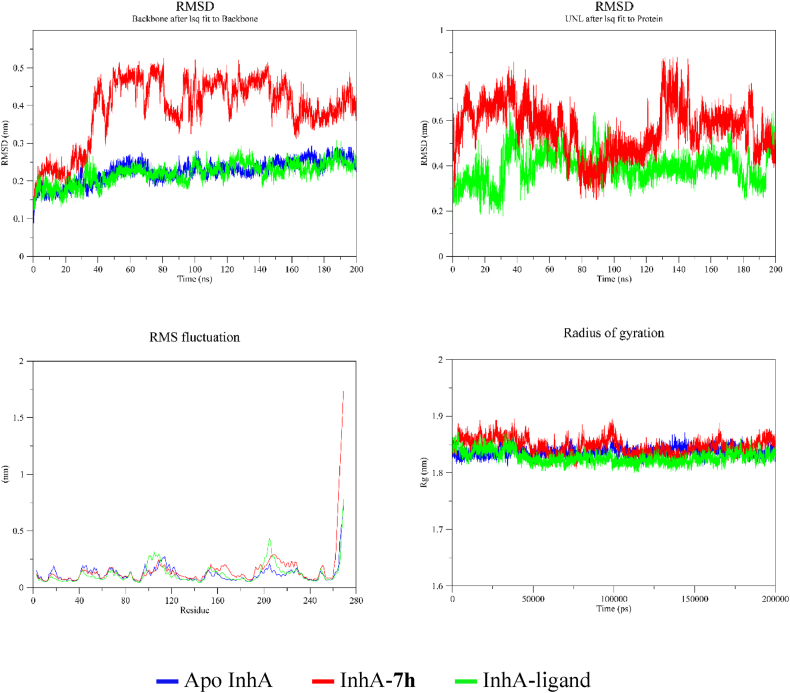
RMSD, RMSF, and Rg plots from the MD simulation trajectories. By the backbone after lsq fit to backbone plot, the RMSD of the protein is shown whereas by the UNL after lsq fit to protein plot the RMSD of the compound is shown.

The relative stabilities of the InhA-**7h** and InhA-ligand complexes procured from the docking and the apo enzyme were investigated through MD simulation. The RMSD, RMSF, Rg, and ligand hydrogen bond plots were drawn from the trajectories and then analysed by comparing them. RMSD of the backbone is used to evaluate structural variations in the whole enzyme structure [63]. The bound ligand containing complex and the apo enzyme exhibited similar RMSD variation trends throughout the 200 ns simulation period. They did not comprise unexpected variations during the simulation period. However, the **7h** containing complex had more variations than the two structures. This complex exhibited a similar course to the other two structures in the first 35 ns. Thereafter, the complex had a rise and variation up to 50 ns. Then, it had less variation up to 68 ns. In the 68–95 ns interval, it had variations. The variations decreased again in the 95–160 ns interval. There were fewer variations next to this time and the average RMSD value also came closer to the other plots (Fig. 4). Hence, the decrease of variations in the last 40 ns implied that the **7h** bearing complex might retain this pattern and could remain stable. Nevertheless, the InhA-**7h** complex's stability was less than the other two structures during the simulation period.

RMSD values of ligands in complex with an enzyme gave a hint about their position inside the binding region of the enzyme [63]. The RMSD plot of the bound ligand and **7h** indicated that the bound ligand was able to exhibit a higher stability in the binding region (Fig. 4). In the first 77 ns interval, the 7h containing complex gave a higher RMSD value. The bound ligand containing complex also had a higher rise in the 31–43 ns interval. The two complexes had similar RMSD values in the 77–130 ns interval with relatively better stability. The bound ligand bearing complex retained the relative stability till 180 ns. However, the **7h** bearing complex had a higher rise in the 130–145 ns. The two complexes had similar variations in the last 20 ns (Fig. 4). The RMSD plot of the two complexes implied that two compounds were able to remain inside the binding region of the enzyme. Together with this, **7h** was able to have higher in and out movements in some intervals. As a stiff rise or fall of the RMSD value of the compound was not observed during the simulation period, **7h** also remained inside the binding region.

RMSF plot is used to evaluate variations in the amino acids of an enzyme during the simulation period [63]. The general RMSF course of the three structures was alike. A significant change was observed in the C-terminal end. Together with this, the InhA-ligand complex had a variation above 0.3 nm in the 198–210 residue interval (Fig. 4). Compactness of an enzyme was measured by using Rg. The Rg allows measuring the effect of ligands on the overall secondary structure of an enzyme [64]. The three structures exhibited similar Rg values with approximately 1.84 nm (Fig. 4). Hence, they were anticipated to possess similar compactness levels.

**Fig. 5:**
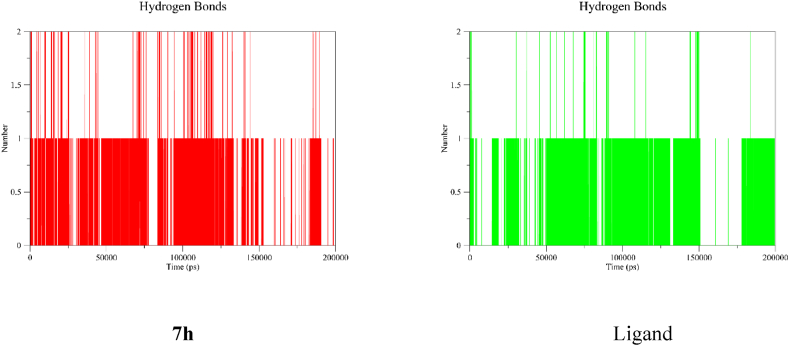
The number of hydrogen bonds for **7h** (in red) and ligand (in green) during the simulation period.

Hydrogen bonds play an important role in maintaining ligands stable inside the binding region of an enzyme [64]. The InhA-**7h** complex had a single hydrogen bond with sparse intermittent two hydrogen bonds. The single hydrogen bond was dense up to 135 ns and in 185–191 ns time intervals. Similarly, the InhA-ligand complex had a single hydrogen bond with sparse intermittent two hydrogen bonds. The single hydrogen bond was dense in 14–34 ns, 42–150 ns, and 178–200 ns time intervals (Fig. 5). Therefore, the two complexes had a single hydrogen bond prominently. In the molecular docking study, the bound ligand and **7h** formed a single hydrogen bonding. The two methods resulted in the same number of hydrogen bonding that confirmed each other. To summarize, the MD simulation study demonstrated that the complexes were stable, and the compounds were able to remain inside the binding region of the enzyme.

### 3.4 MMPBSA computation

MMPBSA energy computations gave values with higher accuracy relative to the other energy computation methods like docking [65]. The MMPBSA computation exhibited that the bound ligand had better affinity to bind to InhA as its binding energy was lower (Table 1). Though the binding energy of **7h** was higher than that of the bound ligand, it was low enough to display potential affinity to bind to the enzyme. The decomposition of the binding energy components showed that van der Waals energy had the highest contribution to the binding energy (Table 1) [66]. The contribution of each residue to the binding energy was calculated with g_mmpbsa [67].

**Table 1:** Binding energy and contributing energies of target-ligand complexes (kJ/mol).

Compounds	Van der Waals Energy	Electrostatic Energy	Polar Solvation Energy	SASA Energy	Binding Energy
Bound ligand	−270.1 ± 8.2	−7.5 ± 1.7	48.5 ± 5.1	−17.9 ± 0.8	−247.0 ± 8.6
**7h**	−230.2 ± 11.9	−17.9 ± 3.8	65.2 ± 6.1	−16.4 ± 0.7	−199.3 ± 10.9

**Fig. 6:**
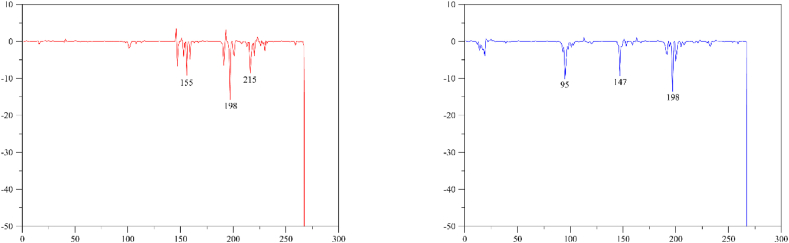
The per residue energy values of InhA-**7h** (in red) and InhA-ligand (in blue) complexes.

In the InhA-**7h** complex Met155, Ala198, and Ile215 amino acids were found to be significant contributors to the binding energy. In the molecular docking study, interaction of the enzyme with **7h** were observed at Ala198 amino acid. Besides, some interactions were near to the residues with relatively low energies (Fig. 6). Similarly, in the InhA-ligand complex Ile95, Met147, and Ala198 had significant contributions to the binding energy. In the molecular docking study, enzyme-ligand interaction was detected at Ala198. Some of the other interactions were found to be near to residues with relatively low energy (Fig. 6).

### 3.5 In silico ADMET prediction

**Fig. 7:**
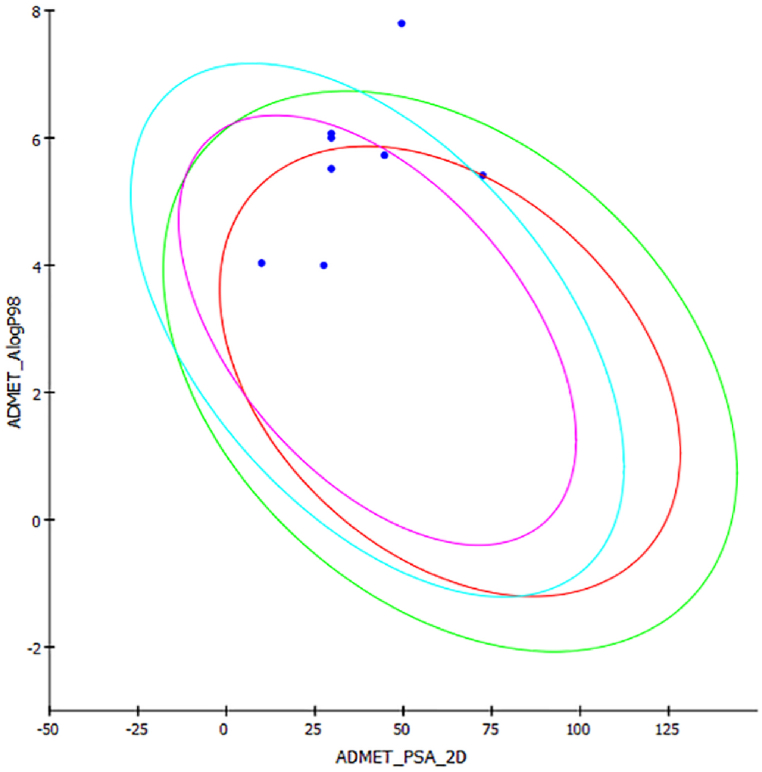
Prediction of the absorption properties through Discovery Studio (absorption 95 in red, absorption 99 in green, BBB 95 in purple, BBB99 in sky blue).

The computational pharmacokinetics investigations demonstrated that most of the synthesized compounds had good ADMET properties. PSA-2D values of all the compounds were predicted to be below one hundred (Table 2). This manifests good oral absorption or membrane permeability for the compounds [68]. Compounds **7b** and **7h** were estimated to have AlogP98 values below five that showed an ideal lipophilic property for them (Table 2) [69]. Compound **7a** had AlogP value somewhat far from five. Thus, its lipophilic property was expected not to be ideal. The rest compounds had AlogP values near to five that demonstrated their lipophilicity property to be within an acceptable range. PSA-2D and AlogP values implied good cell permeability for the compounds except **7a** (Fig. 7) [70].

**Table 2:** Pharmacokinetic predictions of the compounds.

Compounds	AlogP	PSA_2D	PPB	BBB level	Ames mutagenicity	RO5 violations
**7a**	7.795	49.528	4.37	4	Mutagen	1
**7b**	4.032	9.909	2.28	0	Non-mutagen	0
**7c**	5.725	44.659	4.26	0	Non-mutagen	0
**7d**	5.513	29.640	4.46	0	Non-mutagen	0
**7e**	5.999	29.640	5.02	0	Non-mutagen	0
**7f**	6.065	29.640	6.42	0	Non-mutagen	0
**7g**	5.408	72.463	4.06	4	Mutagen	0
**7h**	3.998	27.520	3.54	1	Mutagen	0

The PPB predictions showed that the compounds might bound to plasma proteins (Table 2). This in turn implied low level of binding with blood carrier proteins, which was related with good bioavailability [71].

The possibility of the compounds to cross the BBB was investigated. The result estimated that **7a** and **7g** might have low probability to cross the BBB. In contrast, the BBB might be permeant to the rest compounds (Table 2). From the Ames mutagenicity prediction, compounds **7b**, **7c**, **7d**, **7e**, and **7f** were expected to be non-mutagenic whereas compounds **7a**, **7g**, and **7h** were expected to be mutagenic. The drug-likeness of the compounds was also assessed through Lipinski's rule of five. All the compounds had drug-like properties according to the figures from the SwissADME server prediction (Table 2). None of the compounds, except for compound 7a, violated any of the RO5. Compound **7a** violated just one of the RO5 (Table 2). As a violation was acceptable for a compound to show drug-likeness property, **7a** was also expected to have good absorption or permeability to be used as a drug candidate [72].

## 4 Conclusions

In conclusion, the binding potential of the relatively most active compound (**7h)** to InhA was investigated through molecular docking and MD simulation. The docking study revealed that **7h** had the potential to bind to the enzyme. The MD simulation implied that **7h** could retain its stability in the binding pocket during the simulation period. Moreover, computational pharmacokinetic study exhibited that the synthesized compounds had drug-like properties.

In conclusion, benzimidazole compounds can be further investigated for the treatment of tuberculosis.

## CRediT authorship contribution statement

**Suna Kızılyıldırım:** Supervision, Project administration, Writing – review & editing, Writing – original draft, Visualization, Validation, Methodology, Investigation, Data curation, Conceptualization. **Berfin Sucu:** Writing – original draft, Visualization, Methodology, Investigation, Data curation. **Muhammed Tilahun Muhammed:** Writing – review & editing, Methodology, Formal analysis, Data curation. **Senem Akkoç:** Writing – review & editing, Methodology, Formal analysis, Data curation. **Tuba Esatbeyoglu:** Writing – review & editing, Visualization, Conceptualization, Funding acquisition. **Fatih Ozogul:** Writing – review & editing, Visualization, Conceptualization.

## Data and code availability

All data are within the manuscript.

## Funding

The publication of this article was funded by the Open Access Fund of Leibniz University Hannover.

## Declaration of competing interest

The authors declare that they have no known competing financial interests or personal relationships that could have appeared to influence the work reported in this paper.
